# Acute gluten-induced inflammatory response highlights CCL20 as a potential biomarker for celiac disease

**DOI:** 10.3389/fimmu.2025.1745890

**Published:** 2026-01-12

**Authors:** Sara Gómez-Aguililla, Sergio Farrais, Natalia López-Palacios, Beatriz Arau, Carla Senosiain, Jorge Infante-Menéndez, Ángela Ruiz-Carnicer, Fernando Fernández-Bañares, Nuria González-López, Mar Pujals, Carolina Sousa, Concepción Núñez

**Affiliations:** 1 Laboratorio de Investigación en Genética de enfermedades complejas, Hospital Clínico San Carlos, Instituto de Investigación Sanitaria del Hospital Clínico San Carlos (IdISSC), Madrid, Spain; 2 Aparato Digestivo, Hospital Universitario Fundación Jiménez Díaz, Instituto de Investigación Sanitaria Fundación Jiménez Díaz, Universidad Autónoma de Madrid (IIS-FJD, UAM), Madrid, Spain; 3 Servicio de Aparato Digestivo, Hospital Clínico San Carlos, Instituto de Investigación Sanitaria del Hospital Clínico San Carlos (IdISSC), Madrid, Spain; 4 Department of Gastroenterology, Hospital Universitari Mutua Terrassa, Terrassa, Barcelona, Spain; 5 Centro de Investigación Biomédica en Red de Enfermedades Hepáticas y Digestivas (CIBERehd), Instituto de Salud Carlos III, Madrid, Spain; 6 Servicio de Aparato Digestivo, Hospital Universitario Ramón y Cajal, Madrid, Spain; 7 Departamento de Microbiología y Parasitología, Facultad de Farmacia, Universidad de Sevilla, Sevilla, Spain; 8 Redes de Investigación Cooperativa Orientada a Resultados en Salud (RICORS), Madrid, Spain

**Keywords:** biomarker, celiac disease, gluten challenge, gluten-free diet, protein-inflammatory response

## Abstract

**Background:**

Diagnosis of celiac disease (CD) remains challenging in individuals already on a gluten-free diet (GFD). Although several alternative methods have been proposed, they have limitations. Identifying inflammation-related proteins that rapidly respond to gluten exposure in blood may offer diagnostic alternatives. We aimed to characterize the inflammatory protein response to gluten in patients with CD on a GFD, and to assess the diagnostic potential of candidate biomarkers and their association with clinical symptoms.

**Methods:**

Seventeen patients with CD and 15 non-CD individuals on a GFD (≥ 1 month) consumed 10 g of gluten. Serum, plasma and clinical symptoms were collected at baseline and 4 h post-gluten ingestion to assess changes in 92 inflammation-related proteins. CCL20 levels were also measured by ELISA in plasma from 13 patients with CD and 11 non-CD individuals from the initial cohort, and in an additional group of 28 individuals evaluated for suspected CD, three of whom received a final diagnosis of CD. In 15 patients with CD, the results were compared to those of other diagnostic approaches.

**Results:**

Twelve proteins showed significantly different fold changes following gluten challenge between CD and non-CD groups. Six showed an AUC ≥80%, and CCL20 achieved 81.3-85.7% sensitivity and 88.9-92.7% specificity for CD diagnosis. CCL20 level increases post-gluten challenge were higher in patients with vomiting but were also observed in those with absent or mild symptoms.

**Conclusion:**

Gluten reintroduction triggers alterations in the inflammation-related protein profile of patients with CD. CCL20 emerges as a promising diagnostic candidate, its increase in plasma or serum, with low dependence on symptom presentation, may complement existing diagnostic approaches.

## 1 Introduction

Celiac disease (CD) is an immune-mediated enteropathy triggered by gluten ingestion in genetically predisposed individuals (1). Currently, the only effective treatment is a strict, lifelong gluten-free diet (GFD) (2). CD diagnosis is primarily based on the detection of anti-tissue type 2 transglutaminase antibodies (ATG2) and the confirmation of mucosal damage through intestinal biopsy, except in pediatric cases where non-biopsy criteria may apply (3). Importantly, these diagnostic tests require individuals to be on a gluten-containing diet at the time of evaluation (4). In patients who have already initiated a GFD before a formal diagnosis, guidelines recommend a gluten challenge (GC) (5). However, there is no standardized GC protocol regarding the optimal gluten dose or duration required to reliably induce serological and histological changes. Moreover, many patients are reluctant to undergo a GC due to the risk of symptom relapse, highlighting the need for alternative diagnostic approaches. These would also be valuable when conventional diagnostic tests yield inconclusive results and a GFD is initiated, as occurs in patients with low-grade mucosal lesions or low/negative antibody titers (6).

To address these diagnostic challenges, we previously proposed the study of TCRγδ^+^ intraepithelial lymphocytes (IELs) in duodenal mucosa or the detection of activated gut-homing CD8^+^ T lymphocytes in peripheral blood following a 3-day GC (7, 8). Although both approaches are promising, their clinical applicability may be limited by the need for gluten reintroduction or invasive sampling. Consequently, there is an increasing interest in developing less invasive protocols that require lower gluten doses or avoid re-exposure altogether. In this context, IL-2 has emerged as an attractive diagnostic biomarker (7, 9–13). This discovery arose from investigations into the immunological mechanisms underlying gluten-induced symptoms, in which protein profiling after an oral GC or intradermal gluten peptide exposure in patients with CD revealed an acute IL-2 elevation that was strongly correlated with both the timing and severity of digestive symptoms (10). As most studied patients experienced pronounced symptoms following gluten exposure, further validation in asymptomatic individuals or those with mild symptoms is required to establish the broader applicability of this approach. Moreover, given the low circulating levels of IL-2 in peripheral blood, identification of other upregulated soluble proteins may help overcome technical limitations. Notably, data on immune responses to a single gluten dose in individuals without CD who adhere to a GFD are scarce. Additional research on the effects of gluten reintroduction on circulating proteins is also of interest and may offer insights into CD pathogenesis (9, 10, 13–15).

We aimed to characterize the acute gluten-induced soluble inflammatory protein profile in patients with CD on a GFD, and to assess the diagnostic performance of promising proteins compared with previously proposed alternative biomarkers.

## 2 Materials and methods

### 2.1 Study design

We conducted a multicenter, prospective, quasi-experimental clinical study at four tertiary centers following approval by the respective ethical committees (reference center protocol number: 21/277-E).

### 2.2 Participants and intervention

The initial cohort comprised 32 participants who were included in the screening approach: 17 patients with CD and 15 non-CD controls (9 healthy controls [HC] and 6 participants with suspected gluten-related symptoms [SGRS] (**Supplementary Figure S1**). CD was diagnosed based on positive ATG2 and villous atrophy. All participants followed a GFD for at least one month prior to recruitment. Then, they received a single 10 g dose of powdered gluten (El Granero Integral™; Biogran S.L., Madrid, Spain), in a fasting state, administered with lactose-free liquid yogurt. Peripheral blood samples were collected at baseline (pre-GC) and 4 h after gluten administration (post-GC). Plasma and serum samples were obtained and stored at -80 °C until further analysis.

Additionally, the same protocol was applied to 28 patients who required a GC according to clinical practice. Of these, 3 were ultimately classified as CD and 25 as non-CD. For the validation analysis, this group of 28 participants was combined with a subset of the initial cohort. Specifically, 13 of the original 17 patients with CD and 11 of the original 15 non-CD individuals (9 HC and 2 SGRS) were included. This resulted in a final validation cohort of 52 participants, comprising 16 with CD and 36 non-CD individuals (**Supplementary Figure S1**).

GFD adherence was evaluated by detection of gluten immunogenic peptides (GIP) in urine using an immunochromatographic test (GlutenDetect Urine, Biomedal S.L., Sevilla, Spain) and in feces using ELISA (iVYLISA GIPStool, Biomedal S.L., Sevilla, España) (16, 17).

### 2.3 Screening of inflammation-related proteins

Serum samples from the initial cohort were analyzed using the Olink^®^ Target 96 Inflammation panel (Olink Proteomics, Uppsala, Sweden), which enables multiplex relative quantification of 92 inflammation-related proteins by proximity extension assay (PEA). All samples were analyzed on a single assay plate to avoid inter-plate variability. Data are presented as Normalized Protein Expression (NPX) units, a relative quantification metric on a log_2_-scale. Proteins with detection rates <75% were excluded, following manufacturer’s recommendations. For analytes exceeding this threshold, non-detectable and negative values were removed.

Partial Least Squares Discriminant Analysis (PLS-DA) was performed separately for CD and non-CD groups to evaluate gluten-induced shifts in protein expression. Differences in fold changes (FC), defined as the ratio post-GC NPX/pre-GC NPX values, between groups were analysed using the Mann–Whitney U test. Proteins found to be significantly altered between CD and non-CD groups were retained for subsequent analyses. Spearman correlations calculated within the CD group to assess relationships between FC. The diagnostic performance of individual proteins was evaluated using ROC curve analysis. Optimal thresholds, sensitivity, and specificity were estimated only for proteins with an AUC ≥0.80.

### 2.4 Pathways enrichment analysis

To identify significantly enriched biological processes among the inflammation-related proteins differentially altered between CD and non-CD groups, we performed pathway enrichment analysis using the STRING database (https://string-db.org), focusing on Gene Ontology (GO) Biological Process categories. To distinguish GC-specific pathways in CD from general inflammatory processes, the enriched pathways identified were compared with those obtained from the remaining unaffected inflammation-related proteins. No additional interactors were added to the network to ensure the statistical validity of the enrichment results. An FDR threshold of ≤0.05 was applied to determine significantly enriched GO terms.

### 2.5 CCL20 quantification by ELISA

Absolute plasma CCL20 concentration was measured in the validation cohort using the Elabscience^®^ Human CCL20 ELISA kit (Elabscience, Houston, TX, US), following the manufacturer’s instructions. Samples were diluted 1:2 and analysed in duplicate. The assay lower limit of quantification (LLOQ) was 15.63 pg/mL. Values below this threshold were imputed using the LLOQ for FC estimation. ROC curve analysis was conducted to determine the optimal FC cut-off and corresponding sensitivity and specificity. Spearman’s correlation coefficient was calculated to assess the concordance between FC obtained using PEA and ELISA approaches.

### 2.6 Clinical response

The pre- and post-GC symptom severity was assessed using a 5-point Likert scale. Eight symptoms were assessed: flatulence, abdominal distension, abdominal pain, diarrhea, nausea, vomiting, irritability, and brain fog [adapted from (18)]. Differences between post- and pre-GC clinical scores were analyzed using a two-tailed Wilcoxon test. Additionally, individuals within each group were stratified according to the presence or absence of each clinical symptom, and protein level FC were compared between participants with and without each symptom using a two-tailed Mann-Whitney U test.

### 2.7 Comparison between diagnostic approaches

CCL20 response following GC was compared with previous data obtained from 15 patients with CD using alternative methods: TCRγδ^+^ IELs on a GFD, IL-2 levels following a single gluten dose, and activated gut-homing CD8^+^ T cell responses after a 3-day GC (7).

### 2.8 Statistical analysis

Statistical significance was defined as p ≤0.05. FDR-adjusted values are reported for reference and were not used as the primary significance criterion, except for the pathway enrichment analyses. Statistical analyses were performed using SPSS software (version 15.0). Graphs were generated using the MetaboAnalyst 6.0 platform, STRING database, GraphPad Prism 9, and R software using the ggplot2 package.

## 3 Results

In the initial cohort, 14/17 patients with CD and 12/15 non-CD participants showed correct dietary adherence. In the validation cohort, all individuals adhered correctly, resulting in a total of 16 CD and 36 non-CD participants (**Table 1**). The 3 patients with CD and 3 with SGRS who tested positive for GIP were excluded from all statistical analyses; however, CD patient data were retained to evaluate how dietary adherence influenced the performance of the different diagnostic approaches compared. The final participants included in each comparison are summarized in **Supplementary Figure S1**.

**Table 1:** Characteristics of the participants included in the statistical analyses.

Characteristics	Initial cohort		Validation cohort	
	CD (n=14)	Non-CD (n=12)	CD (n=16)	Non-CD (n=36)
Females (n (%))	11 (78)	9 (75)	14 (82)	25 (69)
Age at inclusion (years)^1^	40.2 ± 3.8	36.1 ± 3.4	41.2 ± 3.7	39.9 ± 2.3
Time on a GFD (months)^2^	32.5 (22, 54.5)	10 (2, 19) (SGRS) 1 (1, 1) (HC)	32.5 (17.5, 65.5)	13 (10, 48) (non-HC) 1 (1, 1) (HC)

Values are expressed as ^1^Mean ± SE and ^2^Median (P25, P75). CD, celiac disease; GFD, gluten-free diet; HC, healthy control; SGRS, suspected gluten-related symptoms.

No significant differences in age or sex distribution were observed between CD and non-CD groups within each cohort. However, the duration of the time on a GFD was significantly longer in the CD group in both cohorts.

### 3.1 Analysis of inflammation-related proteins

Eleven of the 92 inflammation-related proteins were excluded from the analyses due to detection rates <75%. Notably, IL-2 was among the excluded proteins, along with β-NGF, CXCL10, IL-1α, IL-2RB, IL-4, IL-13, IL-24, IL-33, LIF, and TSLP. Seven additional proteins (ARTN, FGF-5, IL-10RA, IL-20, IL-20RA, IL-22RA1, and NRTN) were excluded due to a high number of undetectable or negative values, which reflected very low concentrations. Valid results were obtained for IL-5 in 18 participants (9/14 CD and 9/12 non-CD) and for the remaining 73 proteins in all individuals.

**Figure 1:**
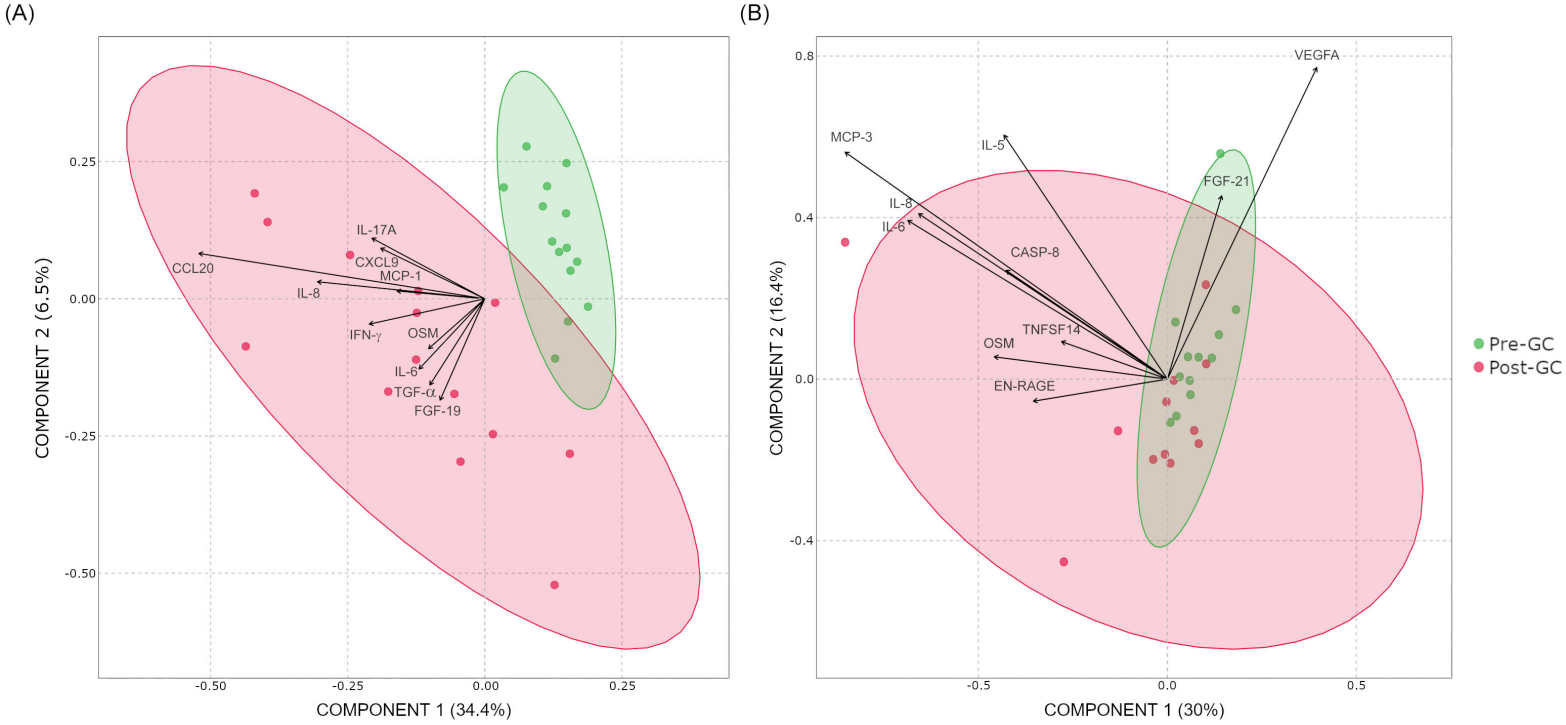
Partial least squares discriminant analysis score plots showing the distribution of samples pre-GC (green) and post-GC (red) in **(A)** patients with celiac disease (CD) and **(B)** non-CD individuals. Arrows represent the proteins with the strongest effect on the separation between groups, with longer vectors indicating greater influence on the model.

Separation of the pre- and post-GC samples using PLS-DA is shown in **Figure 1**. Prior to the GC, CD and non-CD groups displayed quite similar distribution. Following the GC, a clear separation emerged exclusively in patients with CD. This separation was mainly driven by a subset of proteins, with CCL20 showing the highest contribution to the model. In contrast, non-CD samples showed minimal overall separation; however, a few individuals diverged from the main cluster. IL-6 and IL-8 were drivers of sample separation after GC in both CD and non-CD groups.

**Figure 2:**
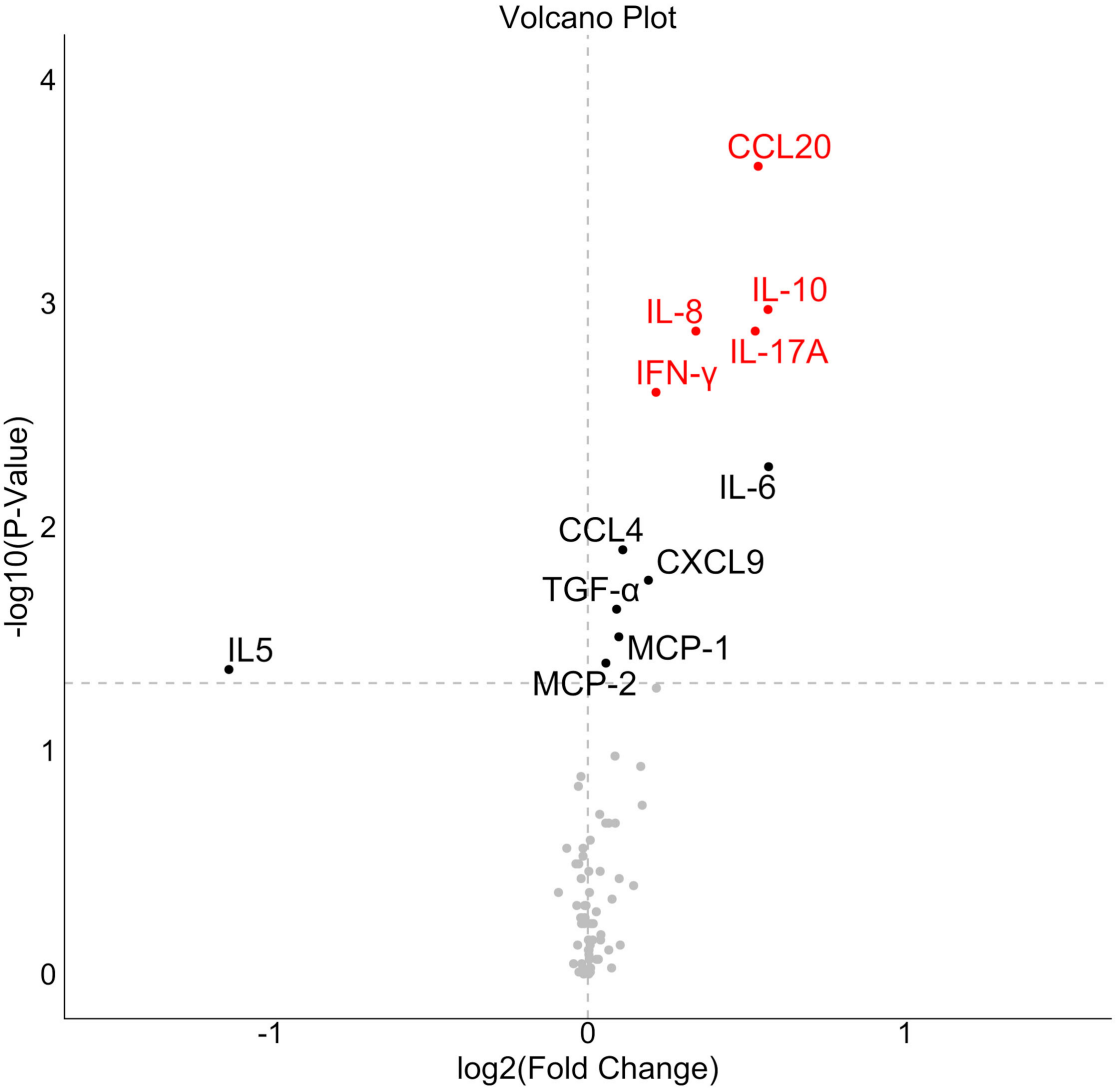
Volcano plot showing proteins differentially affected by gluten challenge in celiac disease (CD) versus non-CD individuals. X-axis represents the median fold change in CD relative to the fold change in non-CD. Analytes highlighted in red indicate statistically significant differences after FDR correction.

Significant differences in FC between CD and non-CD groups were observed for 12 proteins, 5 of which remained significant after FDR correction (**Figure 2**). No correlations were detected between FC and time on a GFD.

### 3.2 Pathway enrichment analysis

**Figure 3:**
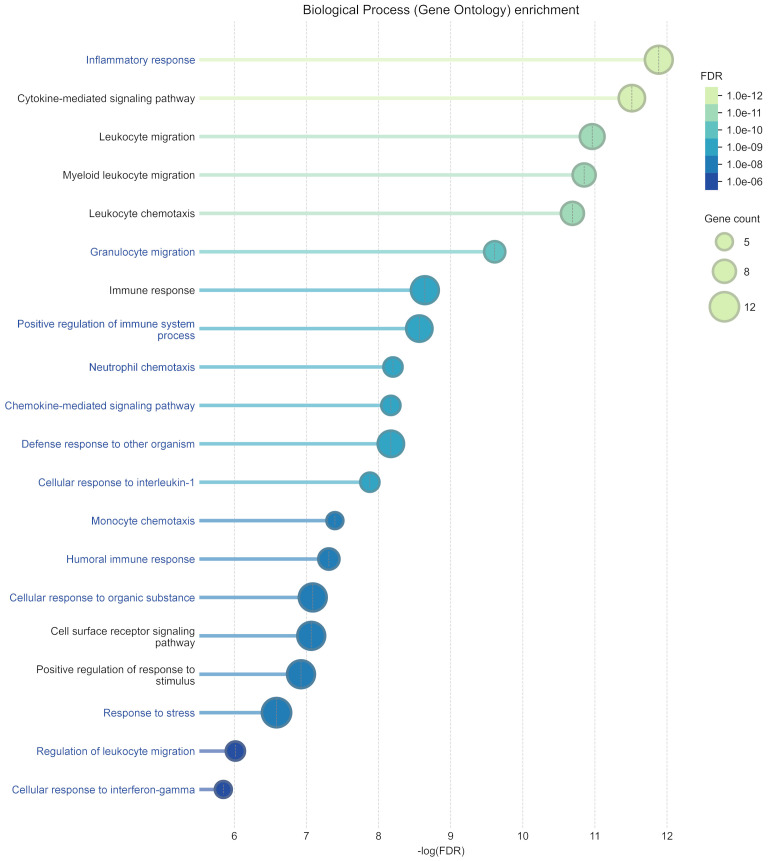
Top 20 enriched pathways considering the 12 proteins with significantly different fold changes between celiac disease (CD) and non-CD individuals after gluten challenge. Pathway names in blue represent pathways uniquely associated with this 12-protein subset, with no overlap with the top 20 pathways identified from the remaining 62 valid proteins in the panel.

**Figure 3** shows the top 20 significantly enriched pathways identified from the 12 proteins differentially altered between groups following GC. When compared with the top 20 pathways obtained from the remaining 62 proteins in the full panel, 13 pathways were found to be exclusive to the differentially altered subgroup, and most were related to cytokine-driven inflammatory responses and immune cell recruitment.

### 3.3 Correlation between proteins

**Figure 4:**
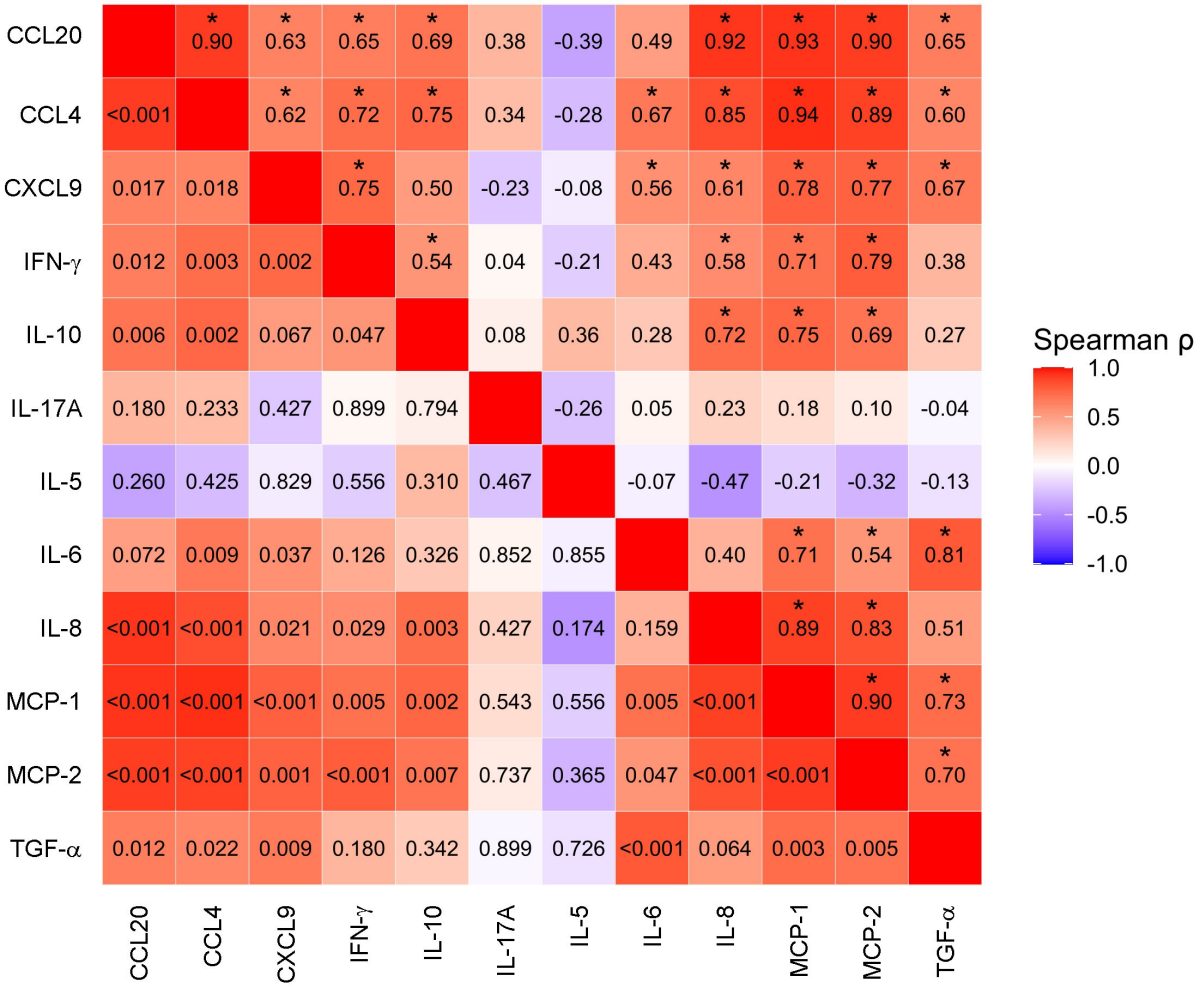
Heatmap showing pairwise correlations in patients with celiac disease (CD) between the 12 proteins with significantly different fold changes between CD and non-CD individuals after gluten challenge. Correlation coefficients (ρ) are displayed above the diagonal, and the corresponding p-values are shown below the diagonal. Asterisks (*) indicate statistically significant Spearman correlation coefficients.

FC values for 10 of the 12 proteins significantly altered between CD and non-CD groups showed significant positive correlations with one another. In contrast, FC values of IL-17A and IL-5 appeared to be independent (**Figure 4**).

### 3.4 Clinical symptoms

**Figure 5:**
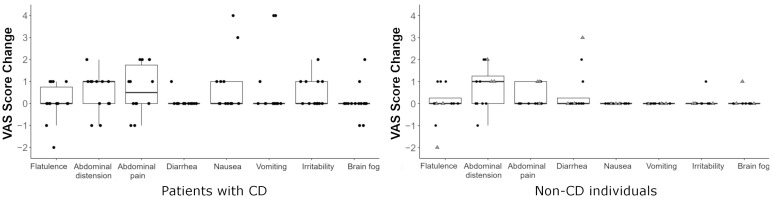
Changes in individual VAS scores for each clinical symptom evaluated in patients with celiac disease (CD, left), and non-CD individuals (right). Grey triangles indicate patients with suspected gluten-related symptoms.

In the initial CD group of 14 patients with negative GIP, 2 patients (14%) reported no change in symptoms, 10 (72%) experienced only mild increases (score change = 1–2), and 2 (14%) reported moderate-to-severe worsening (score change ≥3), particularly nausea and vomiting. In the non-CD group of 12 participants with negative GIP, 4 (33%) participants remained asymptomatic, 7 (58%) reported mild symptom increases (no nausea or vomiting), and 1 (8%) showed a moderate increase (**Figure 5**). In that initial cohort, abdominal distension showed a significant difference in the non-CD group (p=0.047). The global VAS score showed a significant increase post-GC in both groups (CD: p=0.001; and non-CD: p=0.032), although the increase in non-CD was no longer significant when considering only HC (p = 0.125).

**Figure 6:**
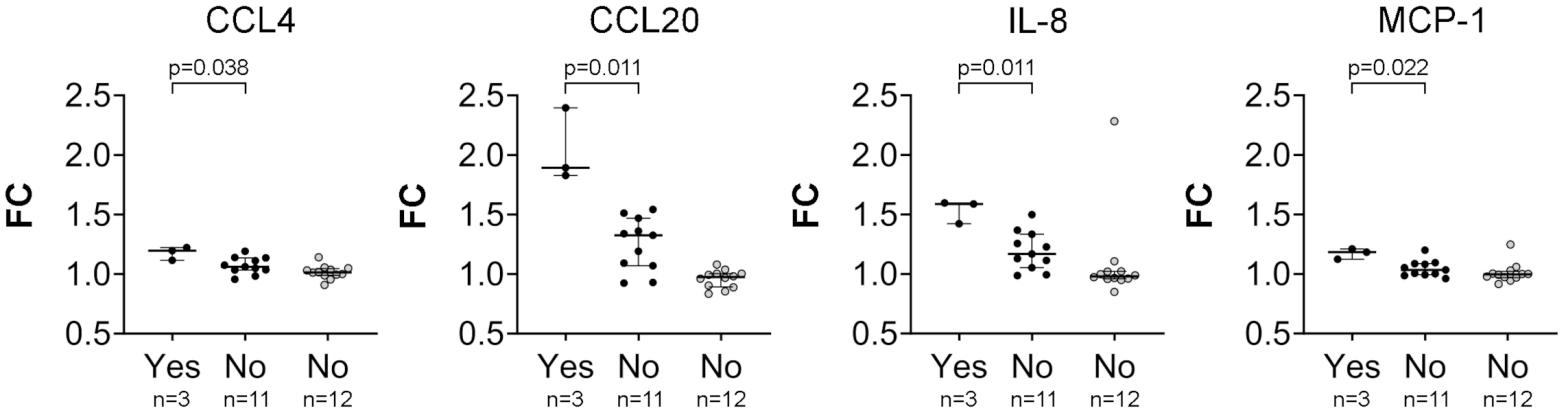
Fold change (FC) values of inflammation-related proteins showing significant differences after gluten challenge according to the presence (Yes) or absence (No) of vomiting. Patients with celiac disease (CD) are represented by black dots, and non-CD individuals by grey dots.

**Figure 7:**
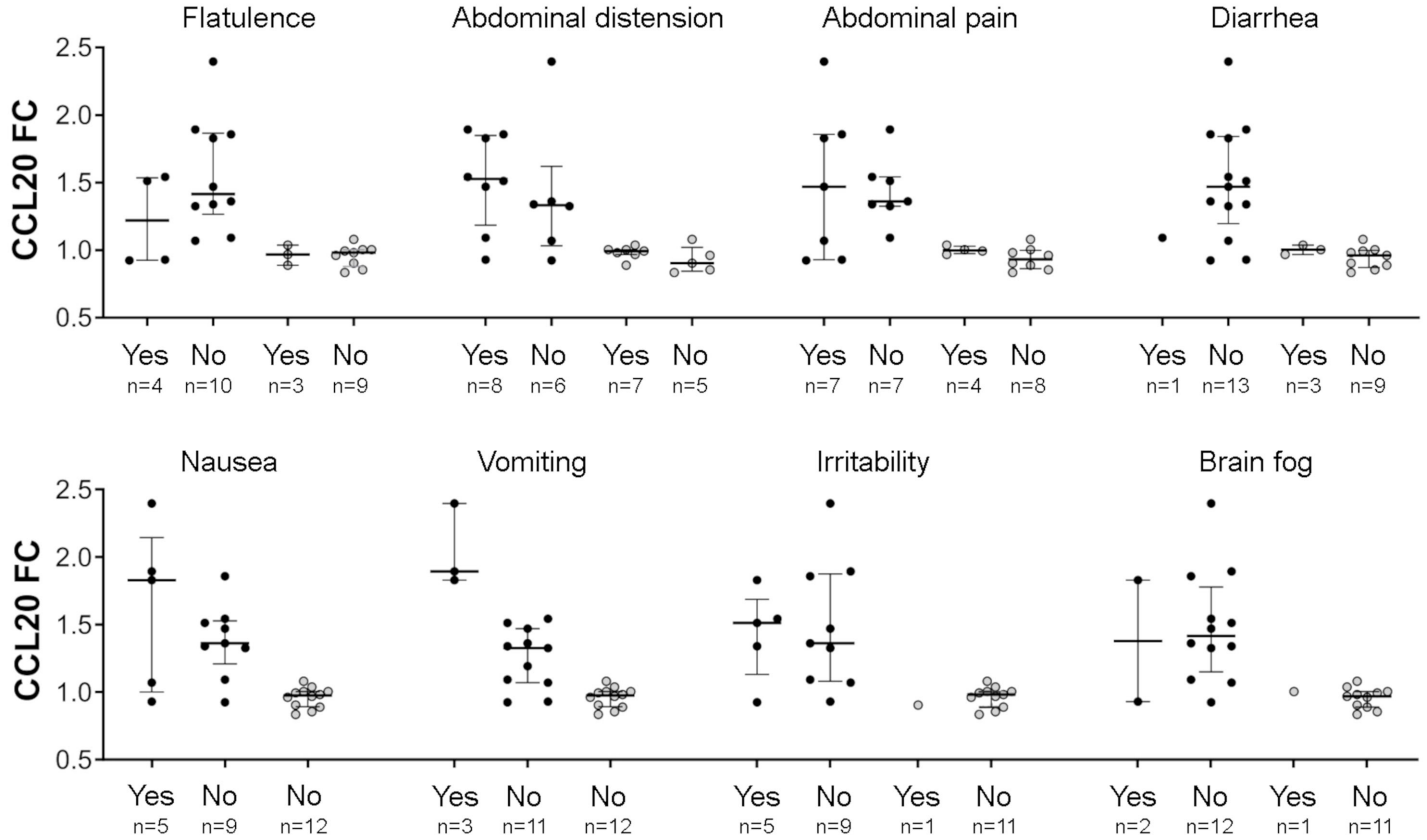
CCL20 fold change (FC) values according to the presence (Yes) or absence (No) of the eight evaluated symptoms. Patients with celiac disease (CD) are represented by black dots and non-CD individuals by grey dots.

When FC values of the 12 proteins differently altered between CD and non-CD groups were compared according to the presence or absence of clinical symptoms, significant differences were observed for vomiting. It was associated with higher FC for CCL20, CCL4, IL-8, and MCP-1 (**Figure 6**). CCL20 FC values stratified by the presence of each evaluated symptom are shown in **Figure 7**.

### 3.5 Biomarker candidates and diagnostic performance

Six of the 12 proteins that differed significantly between CD and non-CD groups showed an AUC ≥80%. FC threshold, sensitivity, specificity, and the corresponding Youden index for each protein are summarized in **Table 2**. CCL20 exhibited the best balance between sensitivity and specificity. Notably, the five proteins that remained significant after FDR correction were also those with the highest AUC values.

**Table 2:** ROC curve results for proteins with AUC ≥80%.

Protein	AUC (%)	FC cut-off	Sensitivity (% [95% CI])	Specificity (% [95% CI])	Youden index
CCL20	89.88	1.080	85.71 (57.19 – 98.22)	92.7 (61.52 – 99.79)	0.784
IL-10	86.31	1.132	85.71 (57.19 – 98.22)	83.3 (51.59 – 97.91)	0.690
IL-17A	85.71	1.156	78.57 (49.20 – 95.34)	83.3 (51.59 – 97.91)	0.619
IL-8	85.71	1.037	85.71 (57.19 – 98.22)	83.3 (51.59 – 97.91)	0.690
IFN-γ	83.93	1.067	71.43 (41.90 – 91.61)	92.7 (61.52 – 99.79)	0.641
IL-6	81.55	1.143	92.86 (66.13 – 99.82)	75.0 (42.81 – 94.51)	0.679

### 3.6 Quantitative validation of CCL20

CCL20 was selected for validation by ELISA, which is suitable for absolute quantification and clinical use. CCL20 plasma concentrations were detectable, at baseline, in 4/17 patients with CD and 7/36 non-CD individuals, and post-GC in 14/17 patients with CD and 8/36 non-CD individuals. All remaining samples were below LLOQ of the assay.

ROC analysis yielded an AUC of 89.1%. The optimal CCL20 FC cut-off was ≥1.22 based on the highest Youden index (0.702), resulting in a sensitivity of 81.3% and specificity of 88.9%. FC values from Olink and ELISA were positively correlated in both groups (CD: ρ=0.752, p=0.003; non-CD: ρ=0.601, p=0.046).

### 3.7 Comparisons between diagnostic approaches

Of the 12 patients with CD and negative GIP who were previously evaluated using alternative methods (TCRγδ^+^ IELs, IL-2, and gut-homing CD8^+^ T cells), 10 were positive in all tests, including CCL20. One patient showed elevated TCRγδ^+^ IELs only, with no CCL20 response, while another tested positive using all alternative methods but lacked a CCL20 response. Regarding the three patients with positive GIP, one showed a positive response in all alternative methods and an increase in CCL20 levels, one had partial positive results (CD8^+^ T cells and TCRγδ^+^ IELs) with an increase in CCL20 levels despite a negative IL-2 result, and the remaining patient tested negative in all three approaches and showed no CCL20 response.

## 4 Discussion

In this study, we assessed the response to a single 10 g dose of gluten in individuals with CD and non-CD controls by quantifying 74 inflammation-related proteins. Of these, 12 showed significantly different responses between groups, and 6 displayed a high AUC, suggesting diagnostic relevance. Importantly, CCL20 emerged as a promising novel biomarker for CD diagnosis in individuals on a GFD. Standard quantitative immunoassays for CCL20 such as ELISA are readily available, further supporting the feasibility of translating these findings into routine clinical practice. The inclusion of patients undergoing evaluation for suspected CD from several centers reflects real-world diagnostic practice and enhances the generalizability of our findings.

GC has been reported to alter the profile of immune-related proteins in patients with CD, independently of sample matrix (serum or plasma) (9, 10, 13, 15). Notably, the early increase of IL-2 at 4 hours post-gluten exposure has been proposed as a diagnostic marker for individuals on a GFD (7, 11, 12), with a recent study supporting its potential application regardless of gluten consumption (12). However, the clinical use of IL-2 remains limited by the need for an ultrasensitive detection platform, owing to its low circulating levels. This drawback is less pronounced for CCL20, which has a higher concentration that facilitates detection. According to our Olink and ELISA results, CCL20 may be more suitable for routine testing. Nevertheless, our results suggest that the performance of conventional ELISA could be improved, as higher-sensitivity methods such as Olink provide more accurate diagnostic parameters.

Cytokine responses have been considered key drivers of symptoms following GC in patients with CD, and are specifically associated with acute symptoms such as nausea and vomiting (10, 13). However, some patients exhibit minimal or no symptoms, which may affect the magnitude or detectability of cytokine responses. Interestingly, a previous study indicates that CCL20 levels appear to be less affected by mild symptomatology (9), supporting its potential as a diagnostic biomarker across a broader spectrum of clinical presentations, including mild or asymptomatic cases. Our cohort reflects this variability, as only 14% of the participants with CD reported moderate or high increase of symptoms after GC. We observed significant associations between cytokine responses and vomiting, with CCL20 showing the strongest effect. Notably, this work evidences that most patients without vomiting also exhibited elevated CCL20 levels, and a similar although weaker pattern was observed for IL-8, which seems to show lower specificity. In contrast, MCP-1 and CCL4 did not appear to be reliable markers in asymptomatic individuals. The low number of patients experiencing vomiting limits definitive conclusions, although this trend is consistent with previous data (9). The other symptoms studied did not seem to influence CCL20 response. This lower dependence on symptom severity constitutes an additional advantage.

The comparison of CCL20 with other previously proposed methodologies (IL-2, gut-homing CD8^+^ T cells or TCRγδ^+^ IELs) does not yield conclusive results, given the high degree of concordance observed. Nevertheless, CCL20 may serve as a complementary diagnostic marker when used alongside other tools, which warrants further investigation. Importantly, CCL20 has been involved in recruitment of gut-homing memory T cells via the co-expression of its main receptor, CCR6 (19), while IL-2 has been described to directly reflect the activation of gluten-specific CD4^+^ T cells (12). This reinforces the complementary diagnostic value of these biomarkers. Notably, the potential role of CCL20 as a biomarker has already been suggested in other autoimmune diseases, including rheumatoid arthritis (20) and vitiligo (21). Moreover, CCL20 and its receptor CCR6 may represent potential therapeutic targets for CD (22).

The immunopathogenesis of CD is primarily driven by the interplay between innate and adaptive immune responses, likely coordinated through a complex cytokine network. This is consistent with the strong correlation observed in our study between the significantly altered proteins, which are mainly involved in immune cell recruitment and inflammatory responses according to pathway enrichment analysis. Exceptions to these correlation patterns included IL-17A and IL-5. Notably, IL-17A has been reported to peak at 2 hours after gluten exposure, potentially preceding the time points analyzed in our study (9), indicating that the lack of correlation should be interpreted with caution. Overall, the cytokine alterations prompted by GC in patients with CD on a GFD are not unexpected and underscore the necessity of lifelong, strict gluten avoidance, given the rapidity of the immune response.

The principal limitation of our study is the relatively small sample size, particularly after stratifying participants into subgroups for symptom-specific analyses. A second limitation relates to CCL20 quantification, as concentrations fell below the lower limit of quantification in some samples, requiring value inference, which may have introduced some uncertainty in FC estimation; importantly, any impact on the overall discriminatory performance of CCL20 was likely minimal. Additionally, different sample matrices were used across platforms, with serum for the Olink analysis and plasma for ELISA validation, but the results showed a strong correlation. Internal assessments using matched samples indicated comparable diagnostic performance, and previous studies have successfully utilized both matrices for similar purposes, supporting the robustness of our findings. Finally, the selection of a 10 g gluten dose for the oral challenge may merit consideration. This dose was chosen based on prior evidence, notably the study by Leonard et al. (11), which compared 2 g and 10 g across several diagnostic approaches and reported stronger responses and greater sensitivity with the higher dose. Given that the 10 g dose is generally well tolerated and that CCL20 remained undetectable in some participants, reducing this dose would likely decrease diagnostic sensitivity.

Despite remarkable advances in the understanding of CD over recent decades, its diagnosis remains challenging in certain contexts. Our findings suggest that CCL20 is a promising biomarker for individuals on a GFD. Given the considerable heterogeneity of the clinical, serological, and histological manifestations of CD, further studies are warranted to explore the utility of CCL20 across the full spectrum of the disease.

## Data Availability

The datasets presented in this study can be found in online repositories. The names of the repository/repositories and accession number(s) can be found below: https://hdl.handle.net/20.500.12530/131642.

## Supplementary material

Supplementary Figure 1 Flow diagram showing participant recruitment, exclusions, and the final sample sizes included in the initial and validation cohorts for the different analyses performed. CD: celiac disease; HC: healthy controls; SGRS: suspected gluten-related symptoms; GC: gluten challenge.
