# Supplementary figures and images for: Acute gluten-induced inflammatory response highlights CCL20 as a potential biomarker for celiac disease
Source: Front Immunol. 2026 Jan 12;16:1745890. doi: 10.3389/fimmu.2025.1745890 (PMC12834126; doi:10.3389/fimmu.2025.1745890)

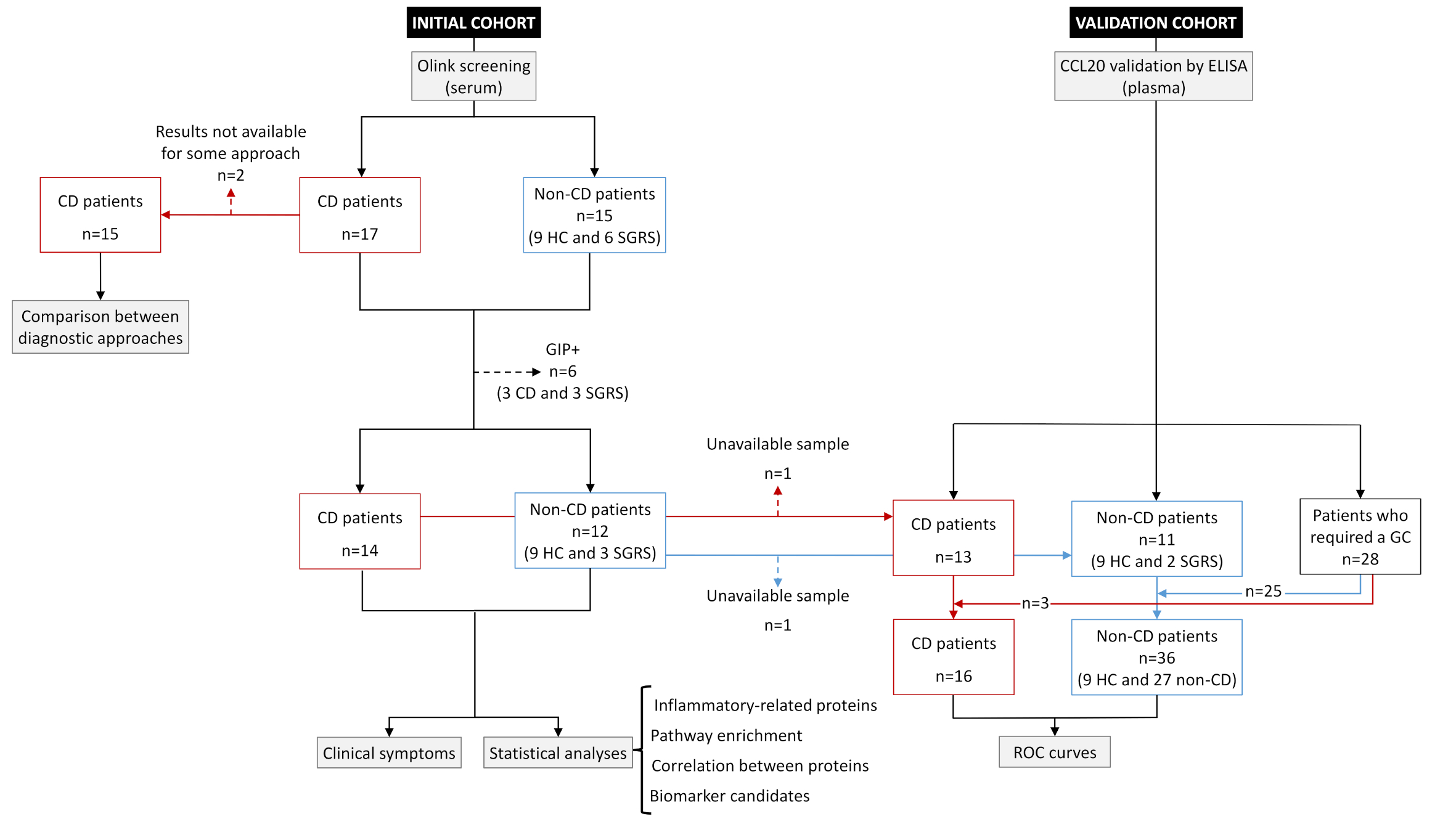

Supplement: Supplementary Figure 1 — Flow diagram showing participant recruitment, exclusions, and the final sample sizes included in the initial and validation cohorts for the different analyses performed. CD: celiac disease; HC: healthy controls; SGRS: suspected gluten-related symptoms; GC: gluten challenge. [file Image1.jpeg]
